# A retrospective study of community-acquired *Clostridium difficile* infection in southwest China

**DOI:** 10.1038/s41598-018-21762-7

**Published:** 2018-03-05

**Authors:** Feng Liao, Wenge Li, Wenpeng Gu, Wenzhu Zhang, Xiaoshu Liu, Xiaoqing Fu, Wen Xu, Yuan Wu, Jinxing Lu

**Affiliations:** 1 Department of Respiratory Medicine, the First People’s Hospital of Yunnan province, 650022 Kunming, China; 2 State Key Laboratory of Infectious Disease Prevention and Control, Collaborative Innovation Center for Diagnosis and Treatment of Infectious Diseases, National Institute for Communicable Disease Control and Prevention, Chinese Center for Disease Control and Prevention, 102206 Beijing, China; 3 Department of Acute Infectious Diseases Control and Prevention, Yunnan Provincial Centre for Disease Control and Prevention, 650022 Kunming, China

## Abstract

To identify the prevalence and characteristics of community-acquired *Clostridium difficile* infection (CA-CDI) in southwest China, we conducted a cross-sectional study. 978 diarrhea patients were enrolled and stool specimens’ DNA was screened for virulence genes. Bacterial culture was performed and isolates were characterized by PCR ribotyping and multilocus sequence typing. Toxin genes *tcdA* and/or *tcdB* were found in 138/978 (14.11%) cases for fecal samples. A total of 55 *C. difficile* strains were isolated (5.62%). The positive rate of toxin genes and isolation results had no statistical significance between children and adults groups. However, some clinical features, such as fecal property, diarrhea times before hospital treatment shown difference between two groups. The watery stool was more likely found in children, while the blood stool for adults; most of children cases diarrhea ≤3 times before hospital treatment, and adults diarrhea >3 times. Independent risk factor associated with CA-CDI was patients with fever. ST35/RT046 (18.18%), ST54/RT012 (14.55%), ST3/RT001 (14.55%) and ST3/RT009 (12.73%) were the most distributed genotype profiles. ST35/RT046, ST3/RT001 and ST3/RT009 were the commonly found in children patients but ST54/RT012 for adults. The prevalence of CA-CDI in Yunnan province was relatively high, and isolates displayed heterogeneity between children and adults groups.

## Introduction

*Clostridium difficile* is an important cause of antibiotic associated diarrhea in patients after hospitalization and antibiotic treatment^1^. *C. difficile* infection (CDI) caused by the toxigenic strains lead to patients with symptoms ranging from asymptomatic colonization to mild diarrhea and life threatening pseudomembranous colitis or rare intestinal obstruction^1,2^. Over the past several years, CDI has surpassed methicillin-resistant *Staphylococcus aureus* as the most common hospital-acquired infection^3^. Although elderly hospitalized patients receiving antibiotics are still the main group with high risk of infection, it is increasingly being recognized that some CDI cases are acquired outside health care facilities, such as younger individuals within community^4,5^. Data from the United States, Canada, and Europe suggest that approximately 20–27% of all CDI cases are community associated (CA-CDI), with an incidence of 20–30 per 100,000 populations^6,7^.

The major pathogenic mechanism of *C. difficile* is the production of enterotoxin A and cytotoxin B, encoded by *tcdA* and *tcdB* genes, which are located, along with surrounding regulatory genes (Paloc)^4,8^. In addition to toxins A and B, parts of *C. difficile* can produce binary toxin, encoded by *cdtA* and *cdtB* genes, and also closely related to the pathogenesis of CDI^8^. Over the past decade, the hypervirulent *C. difficile* isolate, known as restriction endonuclease type BI/pulsed-field type NAP1, toxinotype III, or PCR ribotype (RT) 027, caused several outbreaks and infections with increased incidence and severity in hospitals in 40 states in USA, in all the provinces in Canada and in most European countries since firstly emerged in North American in 2005^9,10^. Other emergent strains of *C. difficile* RT078 also causes severe disease especially associated with CA-CDI^11^.

Although the high rate (~70%) of *C. difficile* colonization in infants <1 year of age^12^, the epidemiology of *C. difficile* in the pediatric population remains poorly understood. Information on CDI in China, especially with a national perspective, is limited. In recent years, many studies, which have been done within single hospital or several hospitals in one place, increased dramatically^13,14^. However, data on CA-CDI in China are extremely rare. According to the definition of CA-CDI, we first investigated the demography, prevalence and molecular characteristics of *C. difficile* among 978 diarrhea cases of outpatients in Yunnan province, southwest China.

## Results

### Clinical features

Among the 978 community-acquired diarrhea patients, 712 cases (72.80%) were children, 266 (27.2%) were adults. The average age was 1.35 ± 2.24 years for children and 48.39 ± 17.72 for adults. The clinical characteristics of two patient groups were different, as shown in Table 1. Except for use antibiotics before hospitals treatment, duration of vomiting and vomit frequency, all the clinical features of two groups were statistical significance (P < 0.05). In children group, the proportion of male, fevers and vomit, the watery stool of fecal property and diarrhea days ≤3 before hospitals treatments were higher than adults; while, the diarrhea days >3 before hospitals treatments and the mucoid, blood stool were more commonly found in adults diarrhea cases. Children patients were enrolled from all the four sentinel hospitals and adults’ cases were collected only from hospital A and D.

**Table 1:** The comparison of clinical features between children and adults in this study.

Parameter	Factors	Age groups				χ^2^	P value
		Children (cases)	%	Adults (cases)	%		
Sex	Male	447	62.78	137	51.5	10.237	0.001
	Female	265	37.22	129	48.5		
Sentinel hospitals	A	143	20.08	85	31.95	448.622	<0.001
	B	411	57.73	0	0.00		
	C	86	12.08	0	0.00		
	D	72	10.11	181	68.05		
Departments	Internal Medicine	295	41.43	266	100	271.590	<0.001
	Pediatric Clinic	417	58.57	0	0.00		
Years	2013	144	20.22	32	12.03	94.470	<0.001
	2014	335	47.05	214	80.45		
	2015	200	28.09	20	7.52		
	2016	33	4.64	0	0.00		
Fever	Yes	295	41.43	24	9.02	92.551	<0.001
	No	417	58.57	242	90.98		
Vomit	Yes	293	41.15	34	12.79	70.030	<0.001
	No	419	58.85	232	87.21		
Fecal property	Watery stool	582	81.74	126	47.37	159.234	<0.001
	Mucoid stool	102	14.33	98	36.84		
	Rice water stool	22	3.09	4	1.50		
	Blood stool	6	0.84	38	14.29		
Use antibiotics before hospitals	Yes	18	2.53	8	3.01	0.172	0.678
	No	694	97.47	258	96.99		
Diarrhea days before hospitals	≤3 days	480	67.42	156	58.65	6.548	0.010
	>3 days	232	32.58	110	41.35		
Diarrhea times/per day	≤5 times	366	51.4	179	67.29	19.861	<0.001
	6–10 times	303	42.56	77	28.95		
	>10 times	43	6.04	10	3.76		
Vomit days before hospitals	≤3 days	277	95.18	30	90.91	1.092	0.296
	>3 days	14	4.82	3	9.09		
Vomit times/per day	≤5 times	232	79.73	28	84.85	0.571	0.450
	>5 times	59	20.27	5	15.15		

### Fecal samples detection

Samples were collected from 2013–2016, and details of the distribution in each year were shown in Table 2. Toxin genes *tcdA* and/or *tcdB* were found in 138/978 cases (Supplementary file 1, S1), and the total positive rate was 14.11% for fecal samples virulence genes (Table 2). Among them, 118 cases (85.51%) were *tcdA*^+^/*tcdB*^+^, 7 cases (5.07%) were *tcdA*^+^/*tcdB*^−^, and 13 cases (9.42%) were *tcdA*^−^/*tcdB*^+^ (S1). In addition, three *cdtA*^+^/*cdtB*^+^ positive cases from hospital D were found in this study and the rest were negative for binary toxins genes (S1). For 138 positive cases, 91 was *tcdC*^+^, 124 were *tcdR*^+^, and 122 were *tcdE*^+^. Among the 138 positive virulence gene samples, 102 cases (73.91%) were from children, only 36 (26.09%) cases from adults. However, the positive samples of children was 14.33% (102/712) and 13.53% (36/266) for adults, showing no statistical significance (χ^2^ = 0.100, P = 0.752). As showed in Table 2, the clinical features of children group were quite similar, such as 46.08% cases had fever and vomit, 83.33% patients had watery stool, 65.69% of cases had diarrhea over 3 days, 93.48% of children patients had vomit more than 3 days, and 76.09% cases with vomit frequency less than 5 times. Furthermore, all these clinical features of children group showed no statistical difference (P > 0.05). While, for adults, the distributions of sentinel hospitals (χ^2^ = 6.250, P = 0.012), fecal property (χ^2^ = 11.721, P = 0.008), duration of diarrhea (χ^2^ = 6.702, P = 0.010) had statistical significance. Compared with children cases, most of the positive cases of adults were from hospital D (86.11%), and the blood stool in adult cases (27.78%) had a relative high proportion. Around 61.11% patients had diarrhea over 3 days. Logistic analysis showed that fever (OR = 2.776, 95%CI = 1.342–5.742, P = 0.006) was the risk factor for positive virulence genes of fecal samples. Other clinical characteristics showed no statistical significance (P > 0.05) (Table 3).

**Table 2:** Comparison of the fecal samples virulence gene and isolation results between children and adults groups

Age groups	Factors	Virulence genes*				χ^2^	P value	Isolation results				χ^2^	P value
		Positive (cases)	%	Negative (cases)	%			Positive (cases)	%	Negative (cases)	%		
	Sex												
Children	Male	68	66.67	379	62.13	0.769	0.380	26	60.47	421	62.93	0.105	0.746
	Female	34	33.33	231	37.87			17	39.53	248	37.07		
Adults	Male	17	47.22	120	52.17	0.306	0.580	5	41.67	132	51.97	0.487	0.485
	Female	19	52.78	110	47.83			7	58.33	122	48.03		
	Sentinel hospitals												
Children	A	13	12.75	130	21.31	7.118	0.068	6	13.95	137	20.48	2.058	0.561
	B	59	57.84	352	57.70			29	67.44	382	57.10		
	C	14	13.73	72	11.80			5	11.63	81	12.11		
	D	16	15.68	56	9.19			3	6.98	69	10.31		
Adults	A	5	13.89	80	34.78	6.250	0.012	0	0.00	85	33.46	5.902	0.015
	B	0	0.00	0	0.00			—	—	—	—		
	C	0	0.00	0	0.00			—	—	—	—		
	D	31	86.11	150	65.22			12	100	169	66.54		
	Departments												
Children	Internal Medicine	41	40.20	254	41.64	0.075	0.784	13	30.23	282	42.15	2.366	0.124
	Pediatric Clinic	61	59.80	356	58.36			30	69.77	387	57.85		
Adults	Internal Medicine	36	100	230	100	—	—	12	100	254	100	—	—
	Pediatric Clinic	0	0.00	0	0.00			0	0.00	0	0.00		
	Years												
Children	2013	14	13.73	130	21.31	5.539	0.136	9	20.93	135	20.18	9.237	0.026
	2014	52	50.98	283	46.39			17	39.53	318	47.53		
	2015	28	27.45	172	28.20			11	25.58	189	28.25		
	2016	8	7.84	25	1.04			6	13.96	27	4.04		
Adults	2013	4	11.11	28	12.17	1.440	0.487	4	33.33	28	11.02	5.983	0.066
	2014	31	86.11	183	79.57			8	66.67	206	81.10		
	2015	1	2.78	19	8.26			0	0.00	20	7.88		
	2016	0	0.00	0	0.00			0	0.00	0	0.00		
	Fever												
Children	Yes	47	46.08	248	40.66	1.059	0.303	19	44.19	276	41.26	0.143	0.705
	No	55	53.92	362	59.34			24	55.81	393	58.74		
Adults	Yes	2	5.56	22	9.57	0.610	0.435	0	0.00	24	9.45	1.246	0.264
	No	34	94.44	208	90.43			12	100	230	90.55		
	Vomit												
Children	Yes	47	46.08	246	40.33	1.193	0.275	28	65.12	265	39.61	10.853	0.001
	No	55	53.92	364	59.67			15	34.88	404	60.39		
Adults	Yes	3	8.33	31	13.48	0.739	0.390	0	0.00	34	13.39	1.842	0.175
	No	33	91.67	199	86.52			12	100	220	86.61		
	Fecal property												
Children	Watery stool	85	83.33	497	81.48	5.174	0.159	37	86.04	545	81.46	1.908	0.592
	Mucoid stool	17	16.67	85	13.93			6	13.96	96	14.35		
	Rice water stool	0	0.00	22	3.61			0	0	22	3.29		
	Blood stool	0	0.00	6	0.98			0	0	6	0.90		
Adults	Watery stool	12	33.33	114	49.57	11.721	0.008	4	33.33	122	48.03	8.091	0.044
	Mucoid stool	12	33.33	86	37.39			3	25.00	95	37.40		
	Rice water stool	2	5.56	2	0.87			1	8.34	3	1.18		
	Blood stool	10	27.78	28	12.17			4	33.33	34	13.39		
	Use antibiotics before hospitals												
Children	Yes	3	2.94	15	2.46	0.082	0.774	2	4.65	16	2.39	0.837	0.360
	No	99	97.06	595	97.54			41	95.35	653	97.61		
Adults	Yes	1	2.78	7	3.04	0.008	0.931	0	0.00	8	3.15	0.390	0.532
	No	35	97.22	223	96.96			12	100	246	96.85		
	Diarrhea days before hospitals												
Children	≤3 days	67	65.69	413	67.7	0.162	0.687	24	55.81	456	68.16	2.804	0.094
	>3 days	35	34.31	197	32.30			19	44.19	213	31.84		
Adults	≤3 days	14	38.89	142	61.74	6.702	0.010	3	25.00	153	60.24	5.866	0.015
	>3 days	22	61.11	88	38.26			9	75.00	101	39.76		
	Diarrhea times/per day												
Children	≤5 times	59	57.84	307	50.33	3.502	0.174	29	67.44	337	50.37	9.686	0.008
	6–10 times	35	34.31	268	43.93			9	20.93	294	43.95		
	>10 times	8	7.85	35	5.74			5	11.63	38	5.68		
Adults	≤5 times	26	72.22	153	66.52	3.766	0.152	11	91.67	168	66.14	3.427	0.180
	6–10 times	7	19.44	70	30.43			1	8.33	76	11.36		
	>10 times	3	8.34	7	3.05			0	0.00	10	22.50		
	Vomit days before hospitals												
Children	≤3 days	43	93.48	234	95.51	0.349	0.555	24	88.89	235	95.53	2.579	0.108
	>3 days	3	6.52	11	4.49			3	11.11	11	4.47		
Adults	≤3 days	3	100	27	90.00	0.330	0.566	0	0.00	30	90.91	—	—
	>3 days	0	0.00	3	10.00			0	0.00	3	9.09		
	Vomit times/per day												
Children	≤5 times	35	76.09	196	80.33	0.372	0.542	21	75.00	213	80.38	0.455	0.500
	>5 times	11	23.91	48	19.67			7	25.00	52	19.62		
Adults	≤5 times	3	100	26	83.87	0.567	0.451	0	0.00	29	85.29	—	—
	>5 times	0	0.00	5	16.13			0	0.00	5	14.71		

^*^The positive virulence gene indicated that at least one of *tcdA* or *tcdB* was positive.

**Table 3:** Logistic regression analysis of clinical features for virulence genes detection and culture results.

Factors	Virulence genes of fecal sample results				Isolation results			
	OR	95%C.I.		P value	OR	95%C.I.		P value
		Lower	Upper			Lower	Upper	
Age group	1.463	0.313	6.835	0.629	1.050	0.110	2.091	0.998
Sentinel hospitals	0.396	0.213	0.734	0.003	0.802	0.344	1.868	0.608
Departments	0.647	0.240	1.745	0.390	0.655	0.205	2.092	0.475
Sex	1.338	0.652	2.748	0.428	0.802	0.333	1.934	0.623
Years	0.690	0.462	1.029	0.068	0.681	0.418	1.111	0.124
Fever	2.776	1.342	5.742	0.006	1.166	0.491	2.767	0.728
Fecal property	0.961	0.495	1.865	0.906	1.083	0.419	2.798	0.870
Use antibiotics before hospitals	0.898	0.713	4.670	0.898	1.077	0.109	10.591	0.950
Diarrhea days before hospitals	0.895	0.454	1.761	0.747	0.806	0.340	1.912	0.625
Diarrhea times/per day	1.280	0.764	2.144	0.349	1.586	0.799	3.146	0.187
Vomit days before hospitals	1.304	0.314	5.421	0.715	0.373	0.084	1.654	0.194
Vomit times/per day	0.767	0.342	1.720	0.520	0.713	0.261	1.948	0.510

### Bacterial culture, toxin profile and PCR ribotyping

**Figure 1:**
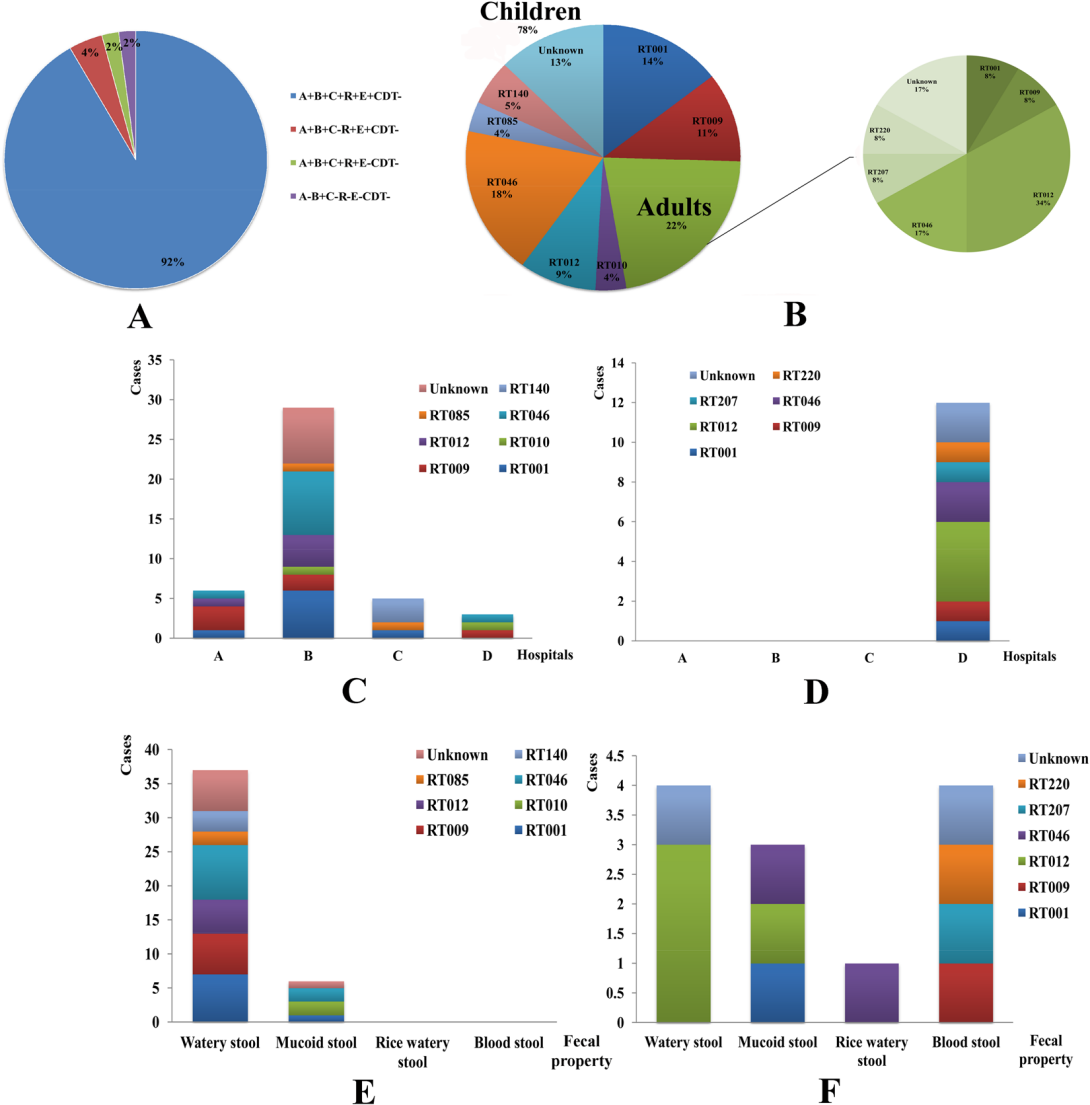
The toxin profiles and PCR ribotyping characters of *C. difficile* between children and adults in this study. (**A**) The toxin profile of isolated strains. (**B**) RT constituent ratio of *C. difficile* strains. Left: children group; right: adults group. (**C**) RT distribution characters of *C. difficile* for different hospitals in children group. (**D**) RT distribution characters of *C. difficile* for different hospitals in adults group. (**E**). RT distribution characters of *C. difficile* for fecal property in children group. (**F**) RT distribution characters of *C. difficile* for fecal property in adults group.

Fifty-five *C. difficile* strains were isolated from 978 fecal samples and the isolation rate was 5.62% (Table 2). For all these isolates, 48 strains (87.27%) were toxigenic, and seven strains were non-toxigenic (S1). Except one *tcdA*^−^/*tcdB*^+^ (YNCD695) strain from pediatric case in hospital B, other strains were *tcdA*^+^/*tcdB*^+^ (Table 2). For the toxigenic *C. difficile*, 45 strains were positive for *tcdC*, 47 were *tcdR*^+^, and 46 were *tcdE*^+^ (S1). The binary toxin genes *cdtA*/*cdtB* were negative for all the isolates (S1). Four toxin profiles were identified: A^+^B^+^C^+^R^+^E^+^CDT^−^, A^+^B^+^C^−^R^+^E^+^CDT^−^ (YNCD169 and 175), A^+^B^+^C^+^R^+^E^−^CDT^−^ (YNCD222) and A^−^B^+^C^−^R^−^E^−^CDT^−^ (YNCD695) (Fig. 1A). This indicated significant diversity in PaLoc area and warranted further deep exploration by amplifying the whole length of the PaLoc region.

Among the 55 strains, 43 (78.18%) were isolated from children, 12 (21.22%) were isolated from adults. The isolation rates between two groups (6.04% for children; 4.51% for adults) showed no statistical difference (χ^2^ = 0.852, P = 0.356). Similar with fecal samples’ results, in children group, 60.47% were male and 67.44% were from hospital B. 44.19% cases had fever, 86.04% patients had watery stool, 55.81% of cases had diarrhea days no more than 3 days. 88.89% of children patients vomited less than 3 days, and 75.00% cases had vomiting frequency no more than 5 times/per day. All these clinical features of children group showed no statistical difference (P > 0.05), as shown in Table 2. However, the distribution of isolated years (χ^2^ = 9.237, P = 0.026), vomit (χ^2^ = 10.853, P = 0.001) and diarrhea times/per day (χ^2^ = 9.686, P = 0.008) showed statistical difference for children group according to bacterial culture results. For adults group, isolation rate of sentinel hospitals (χ^2^ = 5.902, P = 0.015), fecal property (χ^2^ = 8.091, P = 0.044), and diarrhea duration days before hospital treatment (χ^2^ = 5.866, P = 0.015) had statistical significance. All the *C. difficile* strains were isolated from hospital D, 33.33% cases had blood stool, and 75.00% of patients had diarrhea over 3 days (Table 2). Logistic regression analysis showed that all the clinical features had no statistical difference with bacterial culture results (P > 0.05) (Table 3).

A total of 9 RT types were identified, and the details were as follows: 9 isolates of RT001, 7 of RT009, 2 of RT010, 9 of RT012, 12 of RT046, 2 of RT085, 3 of RT140, 1 of RT207, 1 of RT220, and the rest 9 isolates were non-classified by the standard RT library (Fig. 1B). Notably, no hypervirulent RT027 or RT078 were found in this study. 10 (10/43, 23.26%) strains of RT046, 8 (8/43, 18.60%) of RT001, and 6 (6/43, 13.95%) of RT009 were found in children group, accounted for 55.81%. While, in adults group, 4 (4/12, 33.33%) strains of RT012 and 2 (2/12, 16.67%) of RT046 were the most RT types (Fig. 1B). The distribution of RT types had no statistical significance with children and adults groups (χ^2^ = 12.847, P = 0.170). For children patients, RT types showed statistical significance with sentinel hospitals (χ^2^ = 46.717, P = 0.001), departments of hospitals (χ^2^ = 16.766, P = 0.019) and fecal property (χ^2^ = 15.247, P = 0.033). All the RT types could be found in hospital B, except for RT140, RT207 and RT220. Among them, RT046 (8 cases) and RT001 (6 cases) were the most prevalent types, as shown in Fig. 1C. Furthermore, most RT types of patients had watery stool (Fig. 1E). For adults cases, all the clinical features showed no difference with RT types of isolates (P > 0.05). The correlation analysis between RT types and clinical features of patients showed that sentinel hospitals (r = 0.281, P = 0.038), departments of hospitals (r = −0.283, P = 0.036) and fecal property (r = 0.361, P = 0.007) had statistical significance, others had no correlations.

### MLST

**Figure 2:**
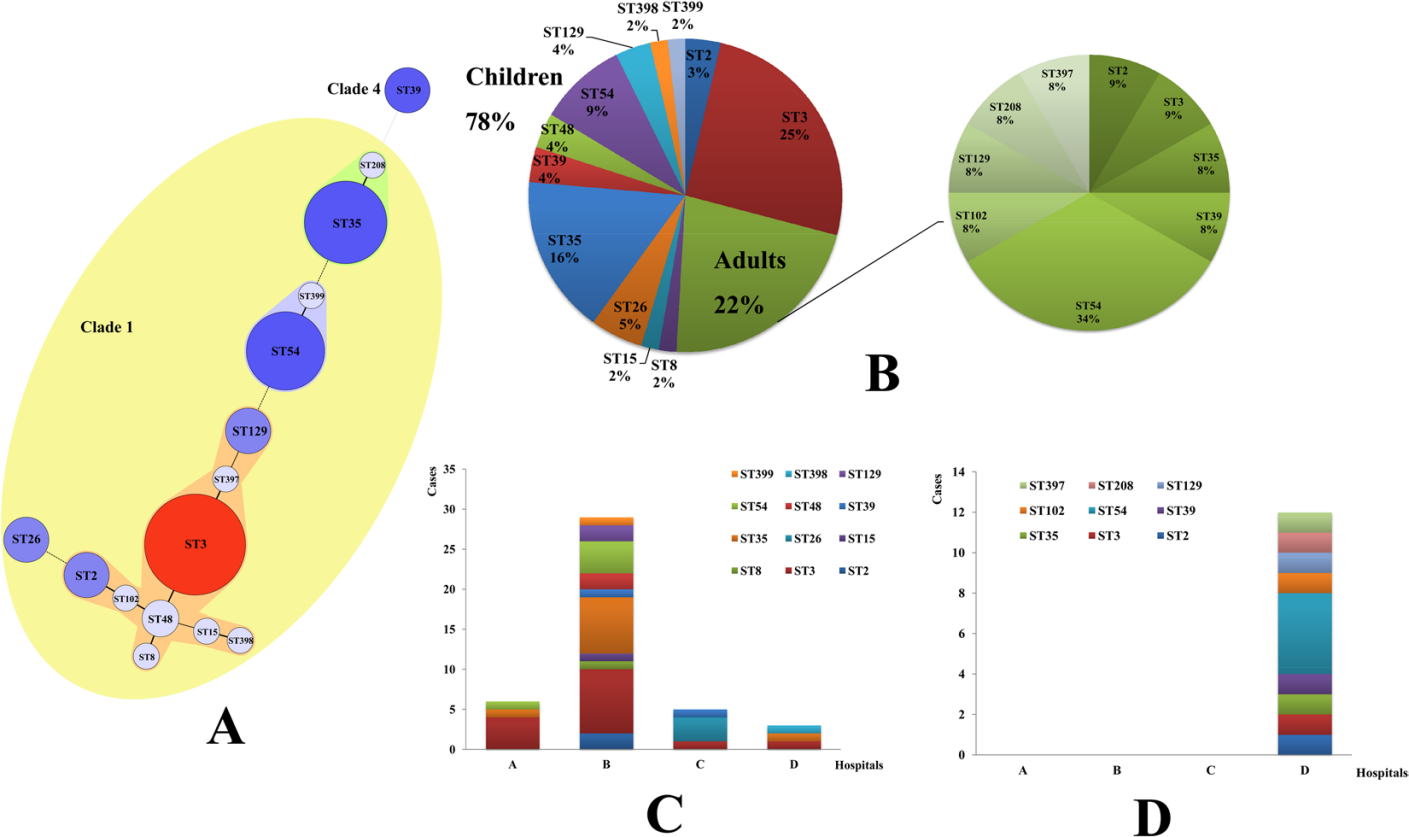
The minimum spanning tree and ST types distribution characters of *C. difficile* between children and adults in this study. (**A**) The minimum spanning tree of isolated *C. difficile* for MLST typing. Each circle corresponds to ST types, the number of which is indicated for the size of circles. The yellow color area belong to the Clade 1. The lines between circles indicate the similarity between profiles (bold, 5 alleles in common; normal, 4 alleles; dotted, ≤3 alleles). (**B**) ST constituent ratio of *C. difficile* strains. Left: children group; right: adults group. (**C**) The distribution characters of *C. difficile* ST types for different hospitals in children group. (**D**) The distribution characters of *C. difficile* ST types for different hospitals in adults group.

MLST results showed that 55 *C. difficile* formed 15 ST types, with a high degree of discrete characteristics (S1). Three novel STs were identified in our study (S1). The toxin profile of ST397 and 399 was the same (positive for *tcdA*, *tcdB*, *tcdC*, *tcdR*, *tcdE*), while for ST398, all these genes were negative. ST3 (15 cases, 27.27%), ST35 (10 cases, 18.18%) and ST54 (9 cases, 16.36%) were the most common ST types, as shown in Fig. 2A. Except ST39 belonging to evolutionary MLST clade 4, all other ST types belonged to the large heterogeneous clade 1 (Fig. 2A yellow area). The distribution of ST types had no statistical significance with children and adults groups (χ^2^ = 19.500, P = 0.147). 14 (14/43, 32.56%) strains of ST3, 9 (9/43, 20.93%) of ST35, and 5 (5/43, 11.63%) of ST54 were mainly types found in children group, accounted for 65.12%. While, in adults group, 4 (4/12, 33.33%) strains of ST54 was the most dominant ST types (Fig. 2B). For children patients, ST types showed statistical significance with sentinel hospitals (χ^2^ = 48.766, P = 0.038). Similar with the RT types results, most of the ST types distributed in hospital B, except ST26, ST102, ST208, ST397 and ST398. Among them, ST3 (8 cases) and ST35 (7 cases) were the most distributed types, as shown in Fig. 2C. Other clinical features of children patients had no difference with MLST types (P > 0.05). For adults cases, all the clinical characteristics showed no difference with ST types (P > 0.05). The correlation analysis between ST types and clinical features of patients showed that sentinel hospitals (r = 0.302, P = 0.025) had statistical significance, others had no correlations.

The ST and RT genotypes had a general agreement, high correlation were revealed in ST35/RT046 (10 cases, 18.18%), ST54/RT012 (8 cases, 14.55%), ST3/RT001 (8 cases, 14.55%) and ST3/RT009 (7 cases, 12.73%), which accounted for 60.00% of all the isolates. The ST and RT genotypes showed statistical significance in both children (χ^2^ = 246.206, P < 0.001) and adults groups (χ^2^ = 72.00, P = 0.014). ST35/RT046 (9/43, 20.93%), ST3/RT001 (8/43, 18.60%) and ST3/RT009 (6/43, 13.95%) were the commonly found genotype profiles in children group, while the most genotype in adults group was ST54/RT012 (4/12, 33.33%).

### Co-infection with other enteric pathogens

In addition to confirming *C. difficile* infection, we also screened the stool samples for other intestinal pathogens^15–17^. A diverse collection of pathogens were detected including diarrheagenic *E. coli*, *Salmonella*, *Shigella*, *Yersinia*, *rotavirus*, *calicivirus*, *astrovirus* and *adenovirus*. Finally, 16 stool samples with positive toxin genes were also found co-exist with one/two other pathogens mentioned above. Details were summarized in supplementary file 1. All 16 samples were positive for *tcdA* and *tcdB*, and negative for *cdtA/B*, except for sample YNCD 654 and 659, which were *tcdA* negative but *tcdB* positive. Sample YNCD550 was detected positive of both diarrheagenic *E. coli* and rotavirus (S1). The age of sample YNCD306 was 45-year-old, and the age of the rest 15 samples were no more than 2-year-old. Three cases (YNCD550, 822, and 831) had co-infection with rotavirus, 13 cases had co-infection with diarrheagenic *E. coli*, and one case (YNCD601) had co-infection with non-typhoidal *Salmonella* (S1). According to the bacterial culture result, 6 strains (patients) were found co-infected with diarrheagenic *E. coli*, non-typhoidal *Salmonella* and *rotavirus*. Among them, one case (YNCD831) had co-infection with *rotavirus*; four cases had co-infection with diarrheagenic *E. coli* and one case (YNCD601) had co-infection with non-typhoidal *Salmonella* (S1). The age of these 6 patients were no more than 2-year-old. Furthermore, two *C. difficile* strains (YNCD399 and YNCD591) were non-toxigenic, others were toxigenic isolates.

Analyzed with the co-infections, 16 cases of positive virulence genes were excluded, 122 cases (12.47%) were CA-CDI for virulence genes detection. For bacterial cultures, 6 cases were excluded for co-infection, and 5 non-toxigenic strains were also excluded, only 44 cases (4.50%) were CA-CDI for isolations.

## Discussion

To our knowledge, this was the first cross-sectional study of CA-CDI in southwest China to address both clinical features and molecular characteristics of *C*. *difficile*. It is known that *C*. *difficile* is found in 30–35% newborns and in 10–15% infants up to 2 years as a commensal bacterium^18–20^, but is potentially harmful for children above two years^5^. Although *C*. *difficile* isolated from an infant under 2 years is normally considered as colonization, we still pay attention to pediatric CDI in this age group if clinical symptoms are typical, or other common intestinal pathogens or physiological diarrhea are excluded. Therefore, we detected toxin genes in all these age groups, and found similar positive isolated rates in both stool samples (14.33% for children, and 13.53% for adults) and isolates (6.04% for children, and 4.51% for adults) between children and adults. The presence of bloody diarrhea was considered as severe CDI for both children and adults^21^. In contrast to studies reported^21–24^, in which a higher proportion of severe CDI in children than that in adult was found, adult patients with bloody diarrhea (38/44 = 86.36%) were much larger than pediatric patients (6/44 = 13.64%) in this study. This might be attributed to lacking of mature toxin binding receptors on epithelial cells, or in complete cellular uptake of the toxins and/or protecting microbiome composition of infants, which leading to insensitive to free *C*. *difficile* toxins in the intestinal tract^22,25^. Antibiotics are usually considered as a risk factor, but not as a prerequisite for developing CDI. A previous study showed that nearly 30% of patients with CA-CDI did not receive antibiotics in the 12 weeks prior to infection^11^. Here, only 2.7% of patients received antibiotics before admission treatment in hospitals.

According to PCR detection of *tcdA/tcdB* of stool samples, 138 samples were positive. Furthermore, 55 isolates were successfully cultured, including 7 non-toxigenic strains which might be missed if only *tcdA/tcdB* positive stool samples were cultured. The average isolation rate (5.62%), and the positive rate (14.11%) for fecal virulence genes in this study was similar with previous studies. A study in United Kingdom illustrated the incidence of CA-CDI increased from 0 to 18 cases per 100,000 persons per year between 1994 and 2004^26^. Data from North America and Europe suggest that 20–27% of all CDI cases are community-associated, with an incidence of 20–30 per 100,000 population^27^. A large study in the Netherlands displayed that the proportion of positive toxin fecal samples (1.5% of 2,443 samples) was remarkably similar to that seen in the UK study by examining all fecal samples using a toxin EIA^28^.

In China, reports on community-acquired CDI were extremely rare. There is only one case of community-acquired CDI, due to a moxifloxacin susceptible RT 027 strain documented in China^29^. Although RT078 was recognized as key pathogen causing CA-CDI, especially in younger adults without hospital contact, but no RT 078 nor 027 were identified here. It was reported that susceptible populations and molecular types of hospital acquired CDI and CA-CDI were distinct. A Europe-wide survey of CDI in 2008 showed that ribotypes 014/020 (15%), 001 (10%), and 078 (8%) were most prevalent; the prevalence of ribotype 027 was 5%^30^. While in another study in North China, ST54 was the dominant type and accounted for 29.2% (66/226) and the other most frequent types were ST3 (25.7%), ST2 (9.7%), ST35 (10.6%), and ST37 (8.4%), but they displayed distinct proportions in diarrhea adults, healthy infant and healthy adults^31^. Similarly, ST3, ST35 and ST54 were the major ST types in our study with different composition rate among diarrhea infants (32.56%, 20.93% and 11.63%) and diarrhea adults (8.33%, 8.33% and 33.33%). However, ST37, previously reported dominant types in China from some studies^32–34^ was not found in this study, which may due to divergence of geography, population, habits and customs. A recent study of hospitalized *C. difficile* infection conducted in Eastern China^13^ showed that ST37/RT017 (16.5%) was the most dominant genotype. RT001 (14.4%), RT012 (13.4%), RT017 (16.6%) and ST2 (11.2%), ST3 (16.3%), ST37 (16.6%), ST54 (12.9%) were predominant. Interestingly, the genotypes profiles in our study were a little bit distinct from that report. ST35/RT046, ST3/RT009 and ST3/RT001 were most common genotypes for pediatric patients, while the most genotype of adult patients was ST54/RT012 in our study.

According to the MLST scheme, almost all isolates clustered in clade 1, and only 3 isolates (ST39) were in clade 4 in this study. The *C*. *difficile* population structure consists of six distinct phylogenetic clades designated 1–5 and C-I^35,36^, and the latter one was highly divergent, entirely nontoxigenic, and potentially a new species or subspecies of *C*. *difficile*^37^. Clade 1 is a highly heterogeneous cluster with toxigenic and nontoxigenic STs and RTs, in North American and Europe^35^. Interestingly, the ST /RT types mentioned above were not the dominant ones in our study, and ST35/RT046, ST3/RT001, ST3/RT009 and ST54/RT012 took highest proportion here. The most notable types in clade 4 is ST 37 (RT017), which has been associated with outbreaks in Europe^38,39^, North America^40^, and Argentina^41^, and is responsible for much of the CDI burden in Asia^42^. However, no ST 37 was identified, leading to focus on clade 4 (containing ST 39 in this study) in China in following research. This further emphasized the importance of national wide surveillance of CDI in China.

This study was the first systemic surveillance for CA-CDI, included children and adults cases in southwest China, in which the prevalence and risk factors were determined. Detailed clinical information and laboratory tests were collected and performed to identify the molecular and epidemiological characteristics of CA-CDI in this area. This study gave us an eye on CA-CDI and caused attention focused on CDI surveillance in China. However, it was indentified several limitations in this study. Firstly, the study was conducted in an urban region that probably showed a poor representation of the potential CA-CDI. Secondly, the diarrhea cases were selected from outpatient cases. But the patients who did not to seek medical advice were not recruited. Thirdly, some of the specific information of *C. difficile* infection probably missed when data collections, such as co-morbidities, exposure to proton-pump inhibitors and histamine-2 antagonists etc. Therefore, further research involved diarrhea cases from urban, rural, outpatient from hospital, and patients from other medical facilities might be done to evaluate the prevalence, clinical features and burdens of *C. difficile* infection.

## Methods

### Patients and case definitions

978 diarrhea cases were collected from January 2013 to March 2016 in four sentinel hospitals in Yunnan Province, southwest China. The fecal samples were continuously collected for each month during that period. The sentinel hospitals were Puji Community Health Service Center (hospital A), Second Affiliated Hospital of Kunming Medical University (hospital B), Kunming Children’s Hospital (hospital C), and First Affiliated Hospital of Kunming Medical University (hospital D). Hospital B to D were tertiary care academic medical center in southwest China, and hospital A was a primary health service center. These four hospitals covered the entire Kunming area. The majority of diarrhea patients came from 12 districts in Kunming, and other cities in Yunnan province, while the rest of patients were from Sichuan or Guizhou provinces in southwest China. The stool specimens from diarrhea patients during the study period were collected and transported to the Yunnan Provincial Center for Disease Control and Prevention. To avoid overrepresentation, only the first stool specimen from each patient was included.

Diarrhea was defined as more than 3 times/per day, accompanied by changes in fecal traits (loose, watery or unformed stool)^43^. Clinical information of patients were collected when they came to hospitals, including gender, age, manifestations *et al*. Patients <18 years was considered as children, ≥18 years were adults. All these cases were defined as community-acquired diarrhea, indicated that the onset of diarrhea symptoms occurring while the patient was outside a healthcare facility, or the patient had no prior stay in a healthcare facility within the 12 weeks prior to symptom onset^44,45^. Therefore, all the *C. difficile* detection positive cases (stool specimens of virulence genes and bacterial isolations) in this study were defined as community-acquired *C. difficile* infection (CA-CDI).

Previous to *C. difficile* detection, the intestinal pathogen spectrums were conducted for all the stool specimens, including diarrheagenic *E. coli*, *Salmonella*, *Shigella*, *Yersinia*, *rotavirus*, *calicivirus*, *astrovirus* and *adenovirus*^15–17^. We analyzed the co-infection results between *C. difficile* and other enteric pathogens mentioned above.

### Detection of virulence genes in fecal samples

DNA extraction was performed on all fecal samples using a fecal sample DNA extraction kit (Tiangen, Beijing) based on the manufacturers’ instruction. The DNA samples were stored at −20 °C and tested for the presence of virulence genes *tcdA*, *tcdB*, *tcdC*, *tcdR*, *tcdE*, *cdtA* and *cdtB*, using primers shown in Table 4. PCR amplification was performed using 20 μl system, 10 μl of Taq premix, 8 μl of water, 0.5 μl of upstream and downstream primers (10 μmol), and 1 μl of template DNA. The amplification reactions were: 94 °C for 5 min; 94 °C 15 sec, 55 °C 30 sec, 72 °C 30 sec, 30 cycles, and finally at 72 °C for 10 min. The products were observed by 1.5% agarose gel electrophoresis and gel imager (Bio-Rad, GelDoc).

**Table 4:** PCR primers used in this study.

Genes	Primers	Sequences (5′–3′)	Amplification length
*tcdA*	tcdA-F	GGACATGGTAAAGATGAATTC	546 bp
	tcdA-R	CCCAATAGAAGATTCAATATTAAGCTT	
*tcdB*	tcdB-F	GTGTAGCAATGAAAGTCCAAGTTTACGC	204 bp
	tcdB-R	CACTTAGCTCTTTGATTGCTGCACCT	
*tcdC*	tcdC-F	TCTCTACAGCTATCCCTGGT	673 bp
	tcdC-R	AAAAATGAGGGTAACGAATTT	
*tcdR*	tcdR-F	CTCAGTAGATGATTTGCAAGAA	473 bp
	tcdR-R	TTTTAAATGCTCTATTTTTAGCC	
*tcdE*	tcdE-F	AGGAGGCGTTATGAATATGA	358 bp
	tcdE-R	TGGTAATCCACATAAGCACA	
*cdtA*	cdtA-F	TGAACCTGGAAAAGGTGATG	375 bp
	cdtA-R	AGGATTATTTACTGGACCATTTG	
*cdtB*	cdtB-F	CTTAATGCAAGTAAATACTGAG	510 bp
	cdtB-R	AACGGATCTCTTGCTTCAGTC	
*16SrRNA*	27 F	AGAGTTTGATCCTGGCTCAG	1465 bp
	1492 R	TACGGCTACCTTGTTACGACTT	
*16–23 S rDNA*	PRB	GTGCGGCTGGATCACCTCCT	variable
	PRBas	CCCTGCACCCTTAATAACTTGACC	
*adk*	adk-P1	GCTATGGTGCCAGCTCTTAC	1500 bp
	adk-P2	GACCAGGTGAGCCTACTATG	

### Bacterial culture

Stool specimens from diarrhea patients were collected using Transwabs (MW&E Ltd., Wiltshire, England), and stored in brain heart infusion (BHI, Oxoid, UK) with 15% glycerol in −80 °C. All faces specimens were inoculated on selective cycloserine-cefoxitin-fructose agar plates (CCFA, Oxoid, UK) with 5% egg yolk after ethanol shock treatment and incubated in an anaerobic jar (80% nitrogen, 10% hydrogen and 10% carbon dioxide) (Mart, NL) at 37 °C for 48 h. *C. difficile* colonies were identified on the basis of their typical morphology and Gram stain as well as the characteristic odour. Suspected colonies were further confirmed by API 20 A (BioMerieux, France) on their biochemical characteristics and 16 SrRNA gene with primer shown in Table 4.

### Bacterial DNA isolation, PCR-ribotyping and toxin gene profiling

Crude bacterial template DNA for toxin profiling was prepared by resuspension of cells in a 5% (wt/vol) solution of Chelex-100 resin (Bio-Rad). All isolates were screened by PCR for the presence of the toxin A (*tcdA*) and toxin B (*tcdB*) genes and the binary toxin (*cdtA* and *cdtB*) genes^46,47^ and the regulating genes of *tcdC*, *tcdR*, and *tcdE*. PCR ribotyping was performed as previously described^48^. PCR ribotyping reaction products were concentrated using a Qiagen Min-Elute PCR purification kit (QIAGEN) and run on a QIAxcel capillary electrophoresis platform (QIAGEN). Visualization of PCR products was performed with QIAxcel ScreenGel software (v1.3.0; QIAGEN). PCR ribotyping banding patterns were identified by comparison of banding patterns with a reference library consisting of a collection of 24 reference strains from the European Centre for Disease Prevention and Control (ECDC), and a collection of 30 isolates from American Type Culture Collection (ATCC). Interpretation of the capillary electrophoresis data (PCR ribotyping banding patterns) was performed using the BioNumerics software package v.7.6 (Applied Maths, Saint-Martens-Latem, Belgium). Isolates that could not be identified with the available reference library were designated with internal nomenclature.

### Multilocus sequence typing

MLST was performed on all recovered isolates using the primers and methods developed by Griffiths *et al*.^49^. Seven housekeeping genes (*adk*, *atpA*, *dxr*, *glyA*, *recA*, *sodA* and *tpi*) were amplified and products were sent for bidirectional sequencing to TaKaRa, Japan. The complete allele sequences were analyzed using DNAStar and MEGA4 software and allele and ST assignments were performed using the *C. difficile* database at pubMLST (https://pubmlst.org/cdifficile). A minimum spanning tree was created using BioNumerics version 7.6. During the experiment, *adk* gene for seven strains couldn’t amplified, and we re-designed the primer (Table 4) based on the Clone Manager Professional Suite 8 software.

### Statistical analysis

Data analysis was performed by IBM SPSS software (version 19.0 for Windows, Armonk, NY). Univariate analysis was performed using the χ^2^ test or Fisher’s exact tests where appropriate. Logistic regression analysis was used to identify independent risk factors. Kruskal-Wallis and χ^2^ test were used to analyze correlation among RTs, STs and clinical features of strains. Odds ratios (ORs), 95% confidence intervals (95%CI), and P values were calculated to assess the differences between groups. Two-sided significance was assessed for all variables, applying a cut-off value of P < 0.05.

### Ethics statements

The human sample collection and detection protocols were carried out in accordance with relevant guidelines and regulations. All experimental procedures were approved by the Ethics Review Committee [Institutional Review Board (IRB)] of National Institute for Communicable Disease Control and Prevention, Chinese Center for Disease Control and Prevention. All adult subjects provided informed consent, and a parent or guardian of any child participant provided informed consent on their behalf.

### Data availability

All data generated or analyzed during this study are included in this published article (and its Supplementary Information files).

## Electronic supplementary material

Supplementary file1
